# Research on the grading method of *Panax notoginseng* slices based on machine vision

**DOI:** 10.3389/fpls.2026.1803945

**Published:** 2026-03-26

**Authors:** Chi Hu, Yongjie Wang, Qinghui Lai, Lu Bai, Wei Su, Jing Zheng, Zhaohui Yao

**Affiliations:** 1 School of Energy and Environment Science, Yunnan Normal University, Kunming, China; 2 Shien-Ming Wu School of Intelligent Engineering, South China University of Technology, Guangzhou, Guangdong, China; 3 Faculty of Modern Agricultural Engineering, Kunming University of Science and Technology, Kunming, Yunnan, China; 4 Kunming Sanxing Conservatory Engineering Co., Ltd., Kunming, China

**Keywords:** grading, machine vision, non-destructive testing, *Panax notoginseng*, saponin content detection

## Abstract

Precise grading is the foundation for improving the safety and consistency of *Panax notoginseng* in clinical applications. This study aims to propose a rapid, non-destructive quality grading method for *Panax notoginseng* based on machine vision and chemometrics. First, high-performance liquid chromatography (HPLC) was employed to determine the Panax Notoginseng Saponins (PNS) content of 143 samples. Based on hierarchical cluster analysis combined with the Elbow Rule, the samples were scientifically categorized into 3-Grade, 5-Grade, and 6-Grade standards. Five machine learning algorithms and six feature selection methods were compared to identify the optimal baseline model, and the Particle Swarm Optimization (PSO) algorithm was introduced to fine-tune the model’s hyperparameters. The final model was evaluated using 10-fold cross-validation, and a bench test was conducted on 50 samples for grading verification. Comparative analysis identified the pearson correlation coefficient combined with CatBoost (COR-CatBoost) as the optimal baseline model across all grading schemes. After hyperparameter fine-tuning with PSO, the final COR-CatBoost-PSO model achieved average classification accuracies of 98.6% ± 0.5%, 88.2% ± 1.2%, and 84.5% ± 1.5% for the 3-Grade, 5-Grade, and 6-Grade standards, respectively, via 10-fold cross-validation. The bench test results showed a 100% classification accuracy of *P. notoginseng*, an average offset of the robotic arm of 1.2 mm, and a single grading process time of 0.8–1.5 s. The results verify the reliability and effectiveness of the proposed rapid, non-destructive quality grading method for *Panax notoginseng*, which can provide technical support for improving the safety and consistency of *Panax notoginseng* in clinical applications.

## 1 Introduction

*P. notoginseng* is an important traditional Chinese medicinal herb in China, with significant medicinal and economic value (Chan et al., 2019; Chen et al., 2022). Precise grading of *P. notoginseng* is crucial for standardizing its industrial production, ensuring consistent medicinal quality, and facilitating the high-quality development of its associated industrial chain within the Chinese medicinal material industry. The traditional quality identification method for *P. notoginseng* primarily relies on its commercial specification grades (based on the number of roots per unit weight). However, this approach lacks support from systematic modernization and pharmacological data. Existing studies have shown no significant correlation between the commercial specifications and the quality of *P. notoginseng* (Dahui et al., 2014; Zhang et al., 2022). Therefore, it is imperative to shift from the traditional Chinese medicinal herb grading method based on “specification grade” to a more comprehensive evaluation method centered on “quality grade” (Xiao et al., 2022).

To date, more than 70 monomeric saponins have been isolated from various parts of *P. notoginseng*, including roots, stems, leaves, flower buds, and seeds. Among these, Panax notoginseng saponins (PNS) are the major active constituents of *P. notoginseng*. PNS is comprised of notoginsenoside R1, ginsenoside Rg1, Re, Rb1, and Rd (Huang et al., 2018; Li et al., 2020; Hanh et al., 2022). Consequently, PNS is widely recognized as a crucial criterion for assessing the intrinsic quality of *P. notoginseng* (Xie et al., 2018). Currently, researchers or testing institutions primarily use high-performance liquid chromatography (HPLC) in the quality assessments of *P. notoginseng* to detect PNS (Guan et al., 2007; Wu et al., 2021). While this method offers high accuracy and reliability, it suffers from of tedious sample pretreatment and lengthy quality control processes, along with high costs. As a result, it is not suitable for industrial-scale or routine rapid quality testing and analysis. To address these limitations, numerous researchers have explored non-destructive testing of *P. notoginseng* quality using chemometric methods. For instance, Yang et al. (2016) employed a method integrating LC-MS fingerprinting and chemometrics to differentiate Panax ginseng, *P. quinquefolium*, and *P. notoginseng*. They analyzed 85 batches of crude drug samples, identified 17 diagnostic chemical markers, and further applied these markers to authenticate the above Panax species in 60 batches of traditional Chinese medicine (TCM) compound preparations. Bian et al. (2022) investigated the applicability of ultraviolet-visible (UV-Vis) diffuse reflectance spectroscopy combined with chemometrics for quantitative analysis of adulterated *P. notoginseng* powder. They constructed a calibration model using partial least squares (PLS), and optimized the model by the Haaland-Thomas F-test, spectral preprocessing, and variable selection strategies. The results showed that the optimal models achieved high prediction accuracy, providing an economical alternative for the detection of adulterated *P. notoginseng* samples. Yang et al. (2018) utilized Fourier transform mid-infrared (FT-MIR) spectroscopy coupled with chemometrics to detect grade-based adulteration of *P. notoginseng* powder, designing 14 different blend ratios of high-grade to low-grade *P. notoginseng*. After spectral preprocessing and characteristic spectral region selection, the support vector machine (SVM) model achieved 100% classification accuracy, enabling the effective identification of adulteration and the detection of blend ratios of 5% and above. Liu et al. (2019) applied near-infrared (NIR) spectroscopy combined with chemometrics to achieve the identification and quantification of *P. notoginseng* and its adulterants. Among the chemometric methods used, partial least squares-discriminant analysis (PLS-DA) and SVM achieved 100% accuracy in species identification; partial least squares regression (PLSR) was confirmed as the optimal calibration method for quantification, and appropriate spectral preprocessing further improved the prediction accuracy of the PLSR model. However, when chemometric methods are employed for the quality detection of *P. notoginseng*, they still face challenges including complex sample pretreatment processes, long detection cycles for a single sample, and the inability to achieve real-time online detection. In view of the aforementioned limitations of existing quality evaluation methods for *P. notoginseng*, there is an urgent need to propose a scientific and efficient grading method to address the current technical bottlenecks.

Machine vision technology simulates the human visual perception by integrating optics, electromechanical technology, and other disciplines to extract, interpret, analyze, and process information from objective images (Olaniyi and Kucha, 2025; Rizvi et al., 2025). It achieves image digitization and coding (Choi et al., 2024b, 2024a). This technology boasts strong versatility, high sensitivity, low cost, and ease of operation, and is widely used in the field of food production (Ordoñez-Araque et al., 2020), and in recent years has begun to be applied in the quality analysis of TCM Slices. Zuo et al. (2025) conducted preliminary differential characterization and identification of *P. notoginseng* from different producing areas based on image recognition and machine learning technologies, and further constructed an image recognition model to achieve accurate classification of *P. notoginseng* origins. Chang et al. (2024) established a large-scale Dendrobium image dataset and proposed an improved YOLOv5 deep learning network for the quality grading of Dendrobium decoction pieces. Huang and Xu (2023) proposed the ResNet101 model for the classification of traditional Chinese medicinal flowers optimized by Bayesian optimization, providing a better solution for the image classification of traditional Chinese medicinal flowers. Given these advantages and the promising application prospects of machine vision technology in the quality analysis of Chinese medicinal materials, this study thus applies machine vision technology to the quality grading of *P. notoginseng* to develop a rapid and efficient grading system.

This study presents a new approach for the grading of *P. notoginseng* Slices based on active ingredient content. Specifically, machine vision was employed to capture cross-sectional images, while HPLC analysis and cluster analysis were combined to establish saponin-based quality grades. By systematically comparing five machine learning algorithms, the COR-CatBoost model was identified as the superior baseline classifier. Furthermore, the Particle Swarm Optimization (PSO) algorithm was utilized to globally optimize the model’s hyperparameters. The main objective of this study was to correlate the external visual features of *P. notoginseng* slices with their intrinsic medicinal quality based on machine vision technology, thereby providing a technical reference for the standardized grading of Chinese medicinal materials.

## 2 Materials and methods

### 2.1 Establishment of quality grading method for *P. Notoginseng*

#### 2.1.1 Instruments and reagents

Image acquisition was performed using a SONY Alpha 6100 mirrorless camera (Sony Corporation, Japan) equipped with a Tamron 17-70 mm F/2.8 (B070) zoom lens (Tamron Optical, Shanghai, China) and a specialized QT-315 HAD camera case (Shenzhen Shengrin Technology, Shenzhen, China). Chromatographic analysis was conducted on a Waters E2695 High-Performance Liquid Chromatography (HPLC) system employing a SunFire C18 column (250 mm × 4.6 mm, 5 μm; Waters Corporation, USA). Sample preparation involved the use of an electronic balance, centrifuge tubes, an ultrasonic cleaner, a slicer, a dryer, a pulverizer, and a 50-mesh standard sieve. Acetonitrile and methanol were of chromatographic grade, while ultrapure water was sourced from Wahaha (Hangzhou, China). All other reagents used were of analytical grade.

#### 2.1.2 Determination of PNS content

**Figure 1:**
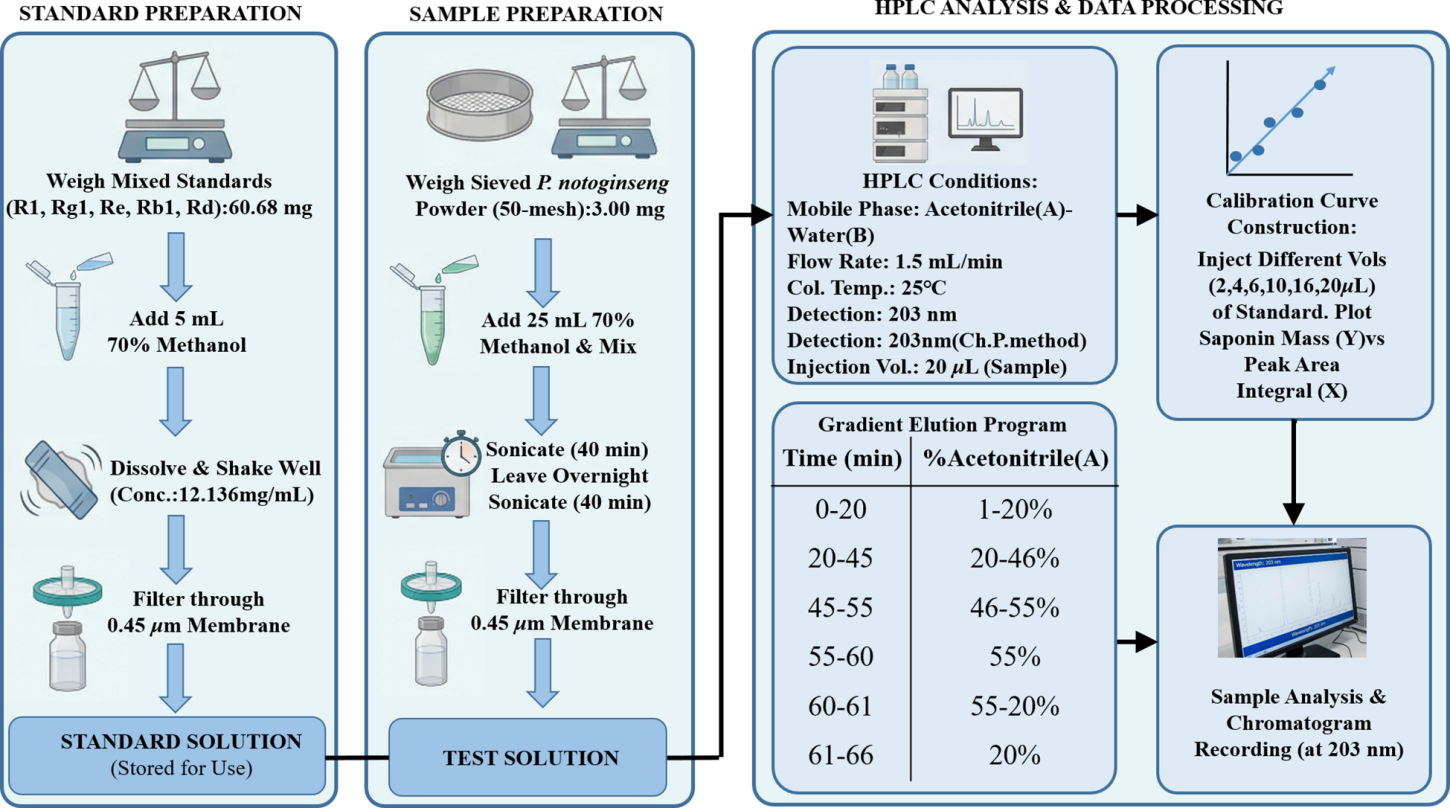
Determination process of PNS content in *P. notoginsengi.* Separation was performed using a gradient elution method with a mobile phase consisting of acetonitrile **(A)** and water **(B)**. The flow rate was maintained at 1.5 mL/min, the column temperature at 25 °C, and the detection wavelength at 203 nm. The injection volume was 20 μL. The specific gradient program was set as follows: 0–20 min, 1–20% A; 20–45 min, 20–46% A; 45–55 min, 46–55% A; 55–60 min, maintained at 55% A; 60–61 min, 55–20% A; and 61–66 min, re-equilibrated at 20% A.

A total of 143 P*. notoginseng* samples were collected from five major producing regions in Yunnan Province: Kunming (n=29), Qujing (n=17), Wenshan (n=34), Honghe (n=48), and Yuxi (n=15). The analytical workflow for PNS determination is illustrated in **Figure 1**.

For the standard solution, 60.68 mg of the PNS reference standard (containing R1, Rg1, Re, Rb1, and Rd) was accurately weighed and dissolved in 5 mL of 70% methanol to obtain a stock concentration of 12.136 mg/mL. For sample preparation, 3.00 mg of sieved *P. notoginseng* powder (50-mesh) was extracted with 25 mL of 70% methanol. The extraction protocol consisted of two 40-min ultrasonic cycles separated by an overnight soaking period. Prior to injection, both standard and sample solutions were filtered through a 0.45 *μ*m membrane.

**Figure 2:**
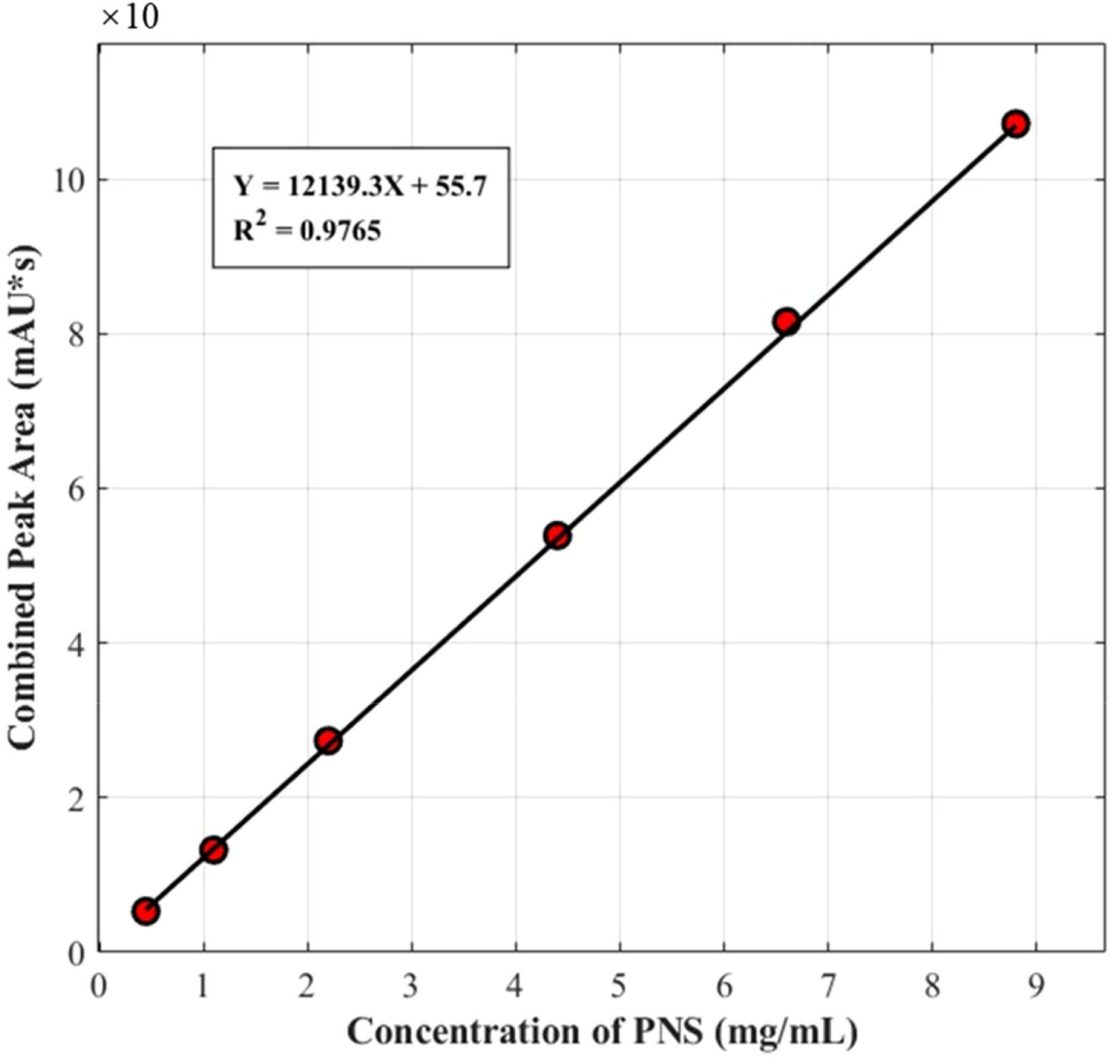
Calibration curve for PNS.

**Figure 3:**
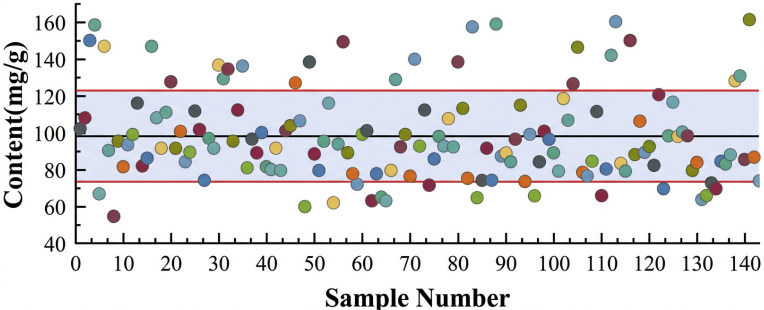
Distribution of PNS content in *P. notoginsengi*.

The calibration curve for PNS was established by plotting the combined peak areas (Y) of R1, Rg1, Re, Rb1, and Rd against the combined concentration (X, mg/mL) of their standard substances. The calibration curve is illustrated in **Figure 2**, with a coefficient of determination (R^2^) of 0.9765. The relatively high R^2^ value indicates a favorable linear relationship, which ensures the accuracy of the subsequent quantitative analysis of the total PNS content. As shown in **Figure 3**, the total saponin content of the 143 P*. notoginseng* samples exhibited significant variation, ranging from 54 to 162 mg/g. The distribution was primarily concentrated in the 80–140 mg/g range, which encompassed over 80% of the samples. The median content was 102 mg/g, with a mean value of 97.3 ± 25.9 mg/g.

#### 2.1.3 Grading of *P. Notoginseng*

Integrating cluster analysis with the elbow method provides a robust methodological framework for establishing quality grading standards for Chinese medicinal materials. In this study, this approach was applied to develop a quantitative quality grading standard for *P. notoginseng*, ensuring an objective and standardized classification system.

Hierarchical Cluster Analysis (HCA)was employed to classify samples based on the similarity of their saponin profiles. The squared Euclidean distance was selected as the distance metric (Equation 1) to amplify differences between samples and enhance cluster separation:

(1) $$d_{i,j}=\sqrt{\sum_{k=1}^{m}\left(x_{ik}-x_{jk}\right)}$$

where $d_{i,j}$ represents the distance between sample *i* and sample *j*, *m* denotes the number of feature dimensions, and *x_ik_* and *x_jk_* are the values of the *k-*th feature for samples *i* and *j*, respectively.

The elbow method was applied to determine the optimal number of clusters (*K*) by evaluating the total within-cluster sum of squared errors (WSS) (Tian et al., 2019; Zhao et al., 2026). The rate of decrease slows significantly once the optimal *K* is reached, forming a characteristic “elbow point”. The total distortion degree *J* is calculated as follows (Equation 2):

(2) $$J={\sum_{K=1}^{K}\sum_{i\in C_{k}}\left|x_{i}-u_{k}\right|}^{2}$$

where *K* is the total number of clusters, *C_k_​* denotes the *k*-th cluster, *x_i_​* represents the *i*-th sample within *C_k_*​, and *μ_k_* is the centroid of cluster *C_k_*​. By plotting *J* against *K*, the elbow points were identified to determine the optimal grading schemes (3-Grade, 5-Grade, and 6-Grade) for *P. notoginseng* quality classification.

### 2.2 Image acquisition and feature extraction

#### 2.2.1 Sample preparation and image acquisition

**Figure 4:**
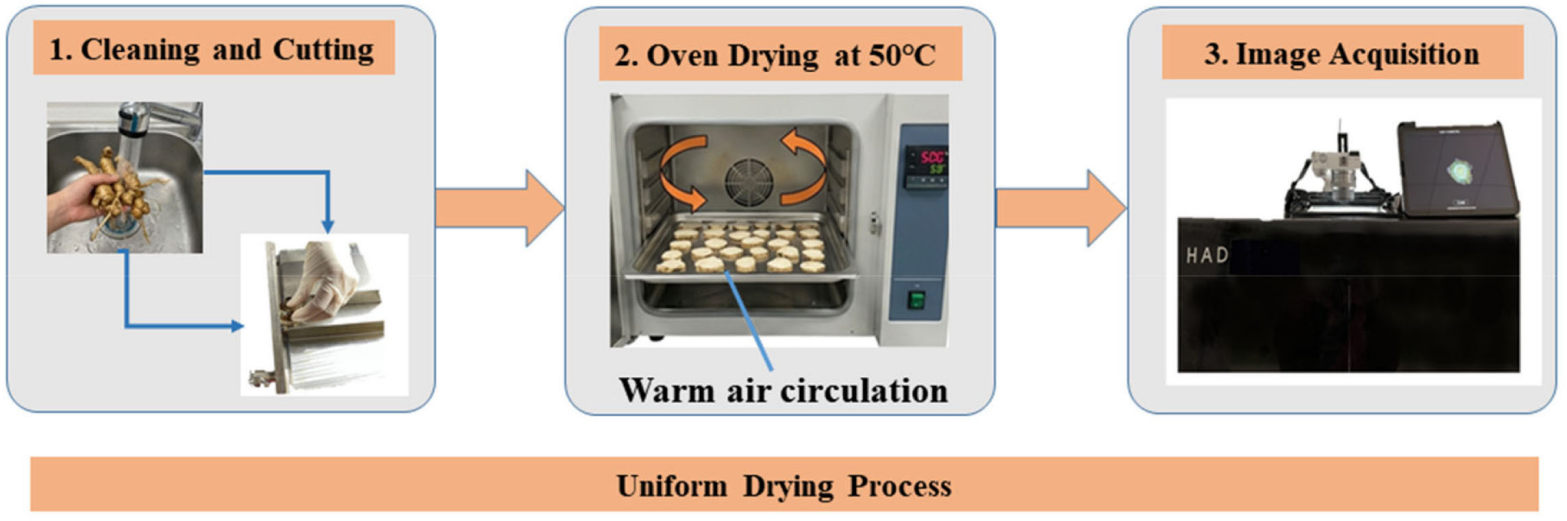
Sample preparation and image acquisition.

A standardized preparation protocol was established to ensure consistency. Fresh *P. notoginseng* samples were cleaned, sliced, and dried in an oven at 50 °C (**Figure 4**). For image acquisition, the dried slices were positioned horizontally on a non-reflective, light-absorbing flocking cloth within a specialized imaging chamber. The imaging system consisted of a camera mounted on a bracket perpendicular to the cross-section of the samples, controlled via a remote shutter to minimize vibration. Illumination was provided by a specialized LED fill light to ensure uniform lighting. To reduce experimental error, each sample was imaged in triplicate.

#### 2.2.2 Extraction of color features

**Figure 5:**
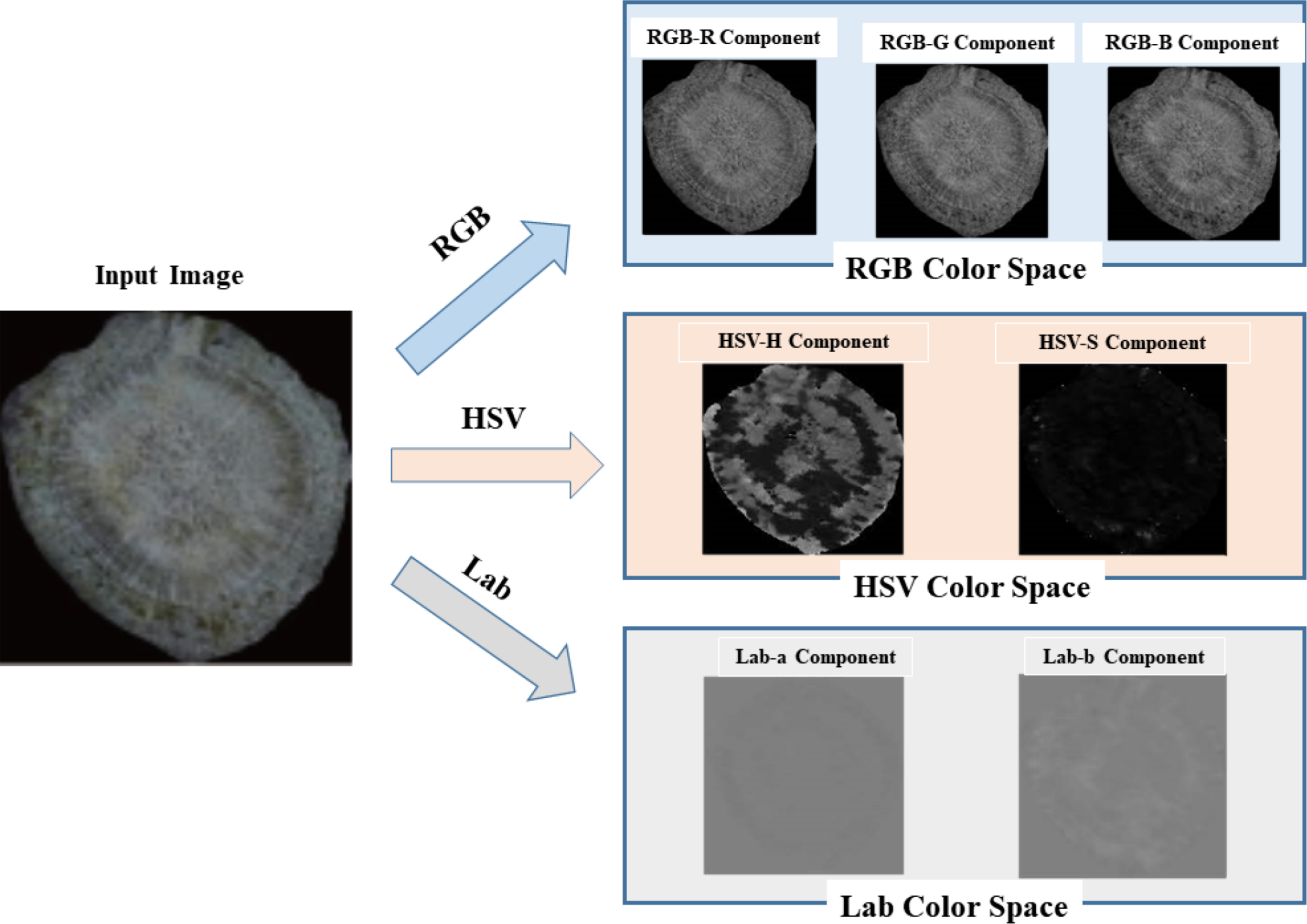
Cross-section image of the slices for component.

To comprehensively characterize the chromatic properties of the *P. notoginseng* cross-sections, images were analyzed using three distinct color spaces: RGB, HSV, and Lab. As the fundamental units for color representation, the R, G, and B channels in the RGB color space can directly reflect the intensity information of the three primary color lights of pixels. In the HSV color space, H (Hue) and S (Saturation) are used to define the category attribute and vividness of colors, respectively, while V (Value) characterizes the brightness dimension feature of images. In contrast, the L (Lightness) in the Lab color space also takes brightness information as the core representation target, and the a and b components focus on describing the deviation attributes of colors. Notably, both the V and L components focus on the key information dimension of “brightness”, which is irrelevant to this study and exhibits significant redundancy. (**Figure 5**).

The transformation from RGB to HSV and Lab is implemented using the following equations (Equations 3, 4). The *H* and *S* are calculated as:

(3) $$H=\arccos\{\frac{\frac{1}{2}\left[\left(R-G\right)+\left(R-B\right)\right]}{{\left(R-G\right)}^{2}+\left(R-B\right)\times \left(G-B\right)}$$

(4) $$S=1-\frac{3}{R+G+B}\min\left(R,G,B\right)$$

The Lab coordinates *a* and *b* are derived using a reference white point ( $X_{n}$=95.047, $Y_{n}$=100.0, $Z_{n}$ =108.883for standard illuminant D65) (Equations 5, 6):

(5) $$a=500\left(f\left(\frac{X}{X_{n}}\right)-f\left(\frac{Y}{Y_{n}}\right)\right)$$

(6) $$b=200\left(f\left(\frac{X}{X_{n}}\right)-f\left(\frac{Z}{Z_{n}}\right)\right)$$

where the function f(t)is defined as:


$$f\left(x\right)=\{\begin{array}{cc} x^{\frac{1}{3}} & t>{\left(\frac{6}{29}\right)}^{3}\\ \frac{1}{3}{\left(\frac{29}{6}\right)}^{2}+\frac{4}{29} & otherwise \end{array}$$

Color features serve as representations of the color distribution information within an image, offering a simple and effective approach to express color features (Wei et al., 2022). Given that color information is primarily concentrated in low-order moments, this study extracted the color moments (mean and variance) of the R, G, B, H, S, a, and b components from the cross-sectional images of *P. notoginseng* slices under the RGB, HSV, and Lab color spaces, resulting in a total of 14 color features. The calculation processes of these features are given in Equations 7 and 8.

(7) $$\mu_{i}=\frac{1}{N}\sum_{j=1}^{N}P_{i}j$$

(8) $$\sigma_{i}={\left(\frac{1}{N}\sum_{j=1}^{N}{\left(P_{ij}-E_{i}\right)}^{2}\right)}^{\frac{1}{2}}$$

where*μ_i_* and *σ_i_* denote the mean and standard deviation of the *i*-th color channel, *N* represents the total number of pixels, and $P_{ij}$ is the value of the *j*-th pixel in the *i*-th channel.

#### 2.2.3 Extraction of texture features

Texture analysis was performed using the Local Binary Pattern (LBP) operator, known for its rotational invariance and computational efficiency (Liu et al., 2017). The LBP value for a central pixel (*x_c_, y_c_*) is calculated by Equations 9 and 10:

(9) $$s\left(x\right)=\{\begin{array}{c} 1,g_{p}\geq {ɡ}_{c}\\ 0,g_{p}<{ɡ}_{c} \end{array}$$

(10) $$LBP\left(x_{c},y_{c}\right)=\sum_{p=0}^{7}s\left(g_{p}-g_{c}\right)2^{p}$$

Where *g_c_* is the grayscale value of the center pixel, and *g_p_* (p= 0, 1,…, 7) represents the grayscale values of the 8 neighboring points.

**Figure 6:**
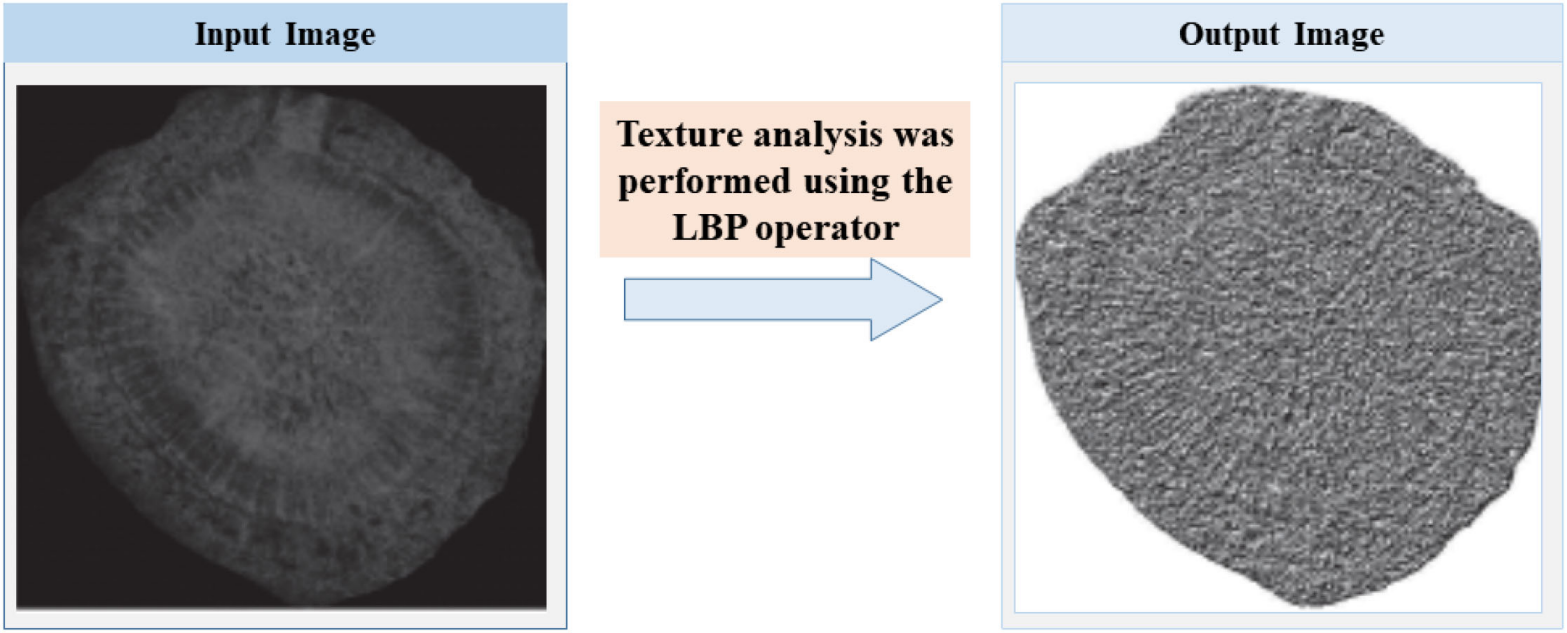
LBP texture features image.

To optimize feature dimensionality, the LBP Uniform Pattern (Ojala et al., 2002) was adopted. This method reduces the 2^8^ = 256 original patterns to 59 distinct feature types (**Figure 6**), which constitute the LBP texture feature vector.

Gabor wavelet transform is recognized for its ability to capture multi-scale and multi-orientation texture information, mimicking the receptive fields of biological visual systems. This makes it ideal for extracting subtle details from *P. notoginseng* cross-sections. The 2D discrete Gabor wavelet transform of an image *f*(x,y) is defined as Equation 11:

(11) $$G_{u,v}(x,y)=\sum_{s}\sum_{t}f\left(x-s,y-t\right)\times g_{u,v}^{*}\left(s,t\right)$$

Where *G_u,v_*(*x,y*) denotes the transformed coefficient, (*x,y*) are the image coordinates, (*x,y*) is the filter mask size, *u* and *v* correspond to scale and orientation parameters, and $g_{u,v}^{*}$ ​is the complex conjugate of the Gabor function.

A bank of 40 Gabor filters (5 scales × 8 orientations) was employed. To characterize texture, the mean energy of the transformed coefficients was calculated for each filter (Equation 12):

(12) $$E_{u,v}\left(x,y\right)=\sum_{x}\sum_{y}\left|Gu,v\left(x,y\right)\right|$$

This yields a 40-dimensional Gabor texture feature vector representing multi-scale and multi-orientation characteristics.

#### 2.2.4 Dataset construction

Given the high intra-class diversity and inter-class similarity of *P. notoginseng* cross-sections, a multi-feature fusion strategy was implemented, and the final feature set integrated three types of features: 14 color moments (Mean and Variance) as Color Features derived from RGB, HSV, and Lab spaces, 59-dimensional Uniform LBP features as LBP Texture Features capturing local structural information, and 40-dimensional mean energy values as Gabor Texture Features extracted via Gabor wavelet transform (5 scales and 8 orientations), which generated a comprehensive dataset and provided a basis for subsequent machine learning models.

### 2.3 Construction of grading model

#### 2.3.1 Sample balancing

**Figure 7:**
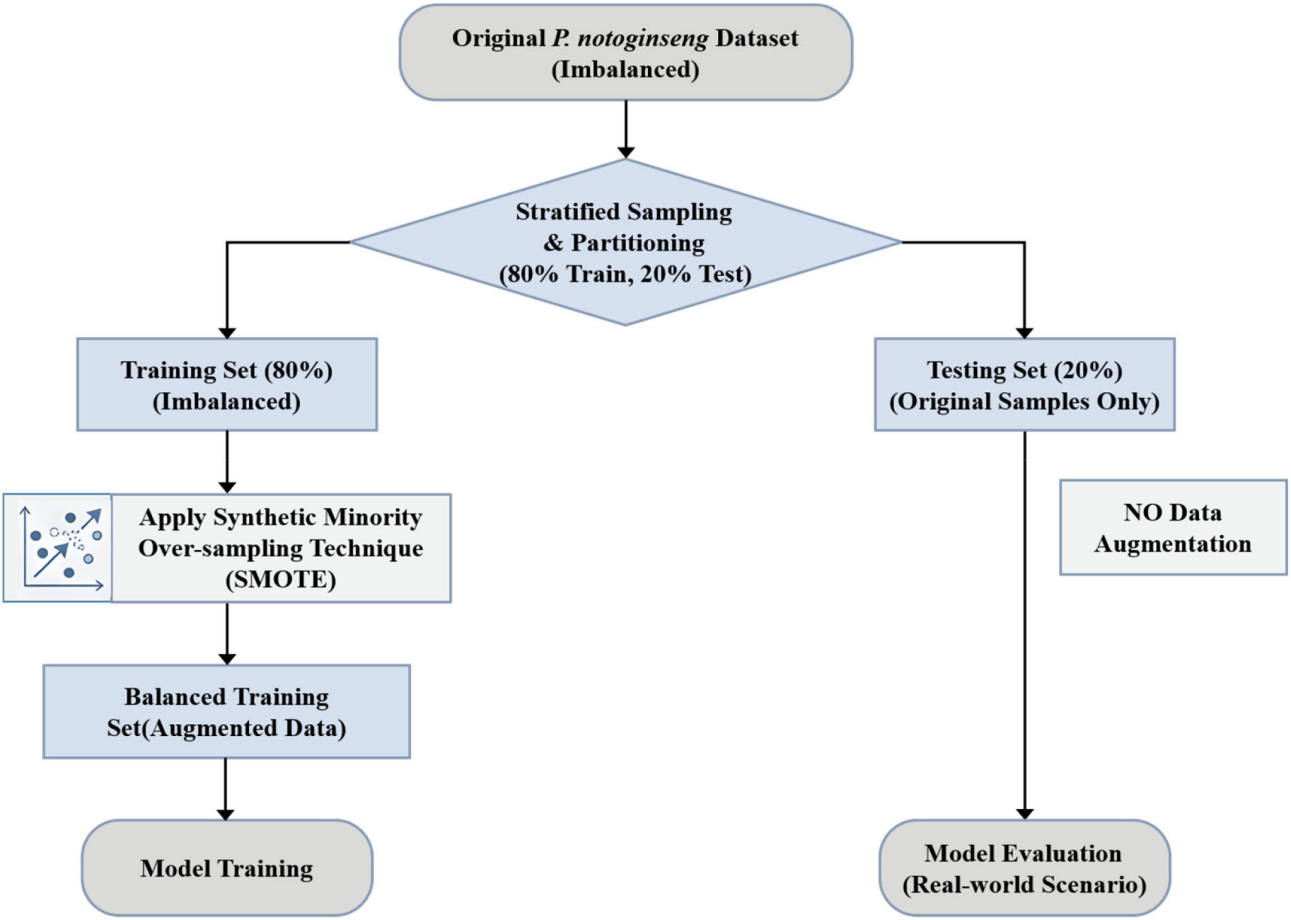
Detailed flowchart of the SMOTE data augmentation process.

**Figure 8:**
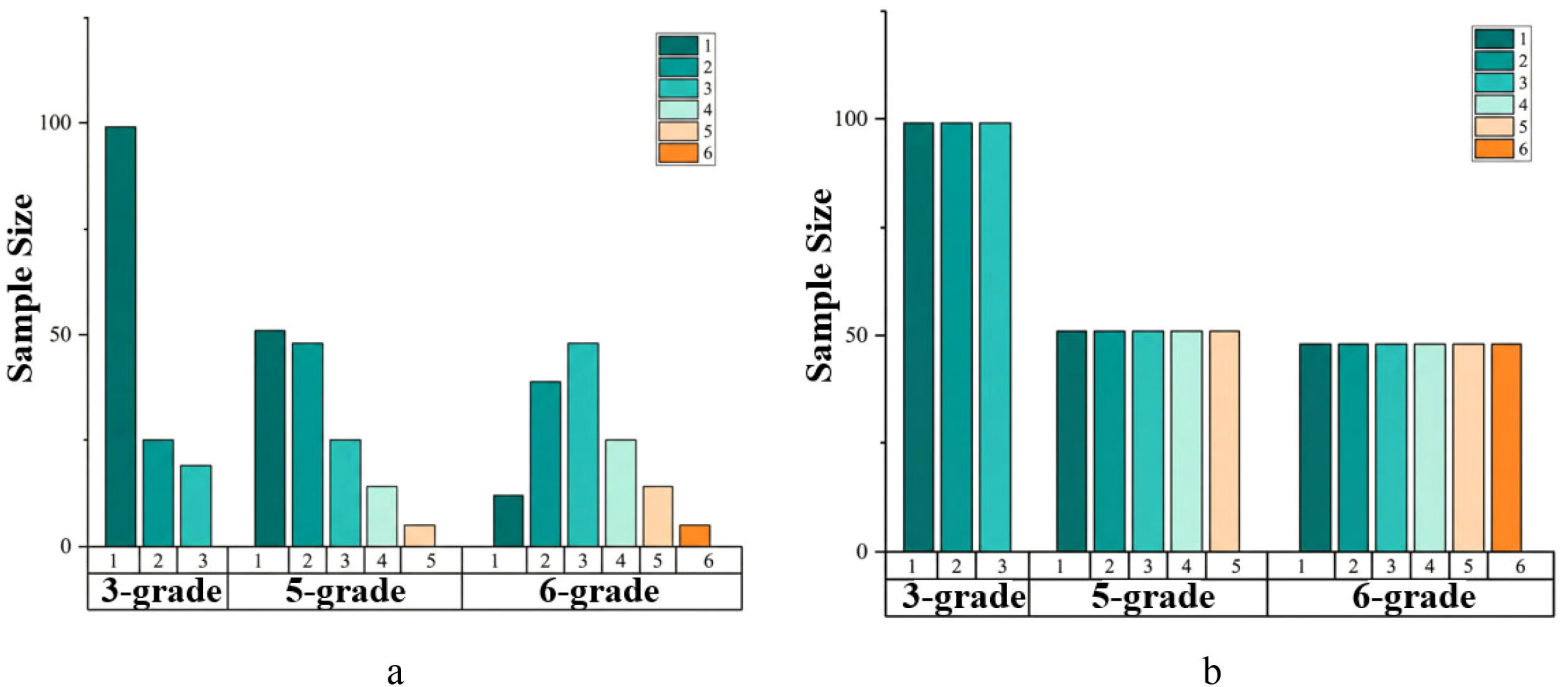
Size of samples contained in each grade before and after sample equalization. **(a)** Before sample equalization. **(b)** After sample equalization.

Prior to model training, addressing class imbalance within the dataset is crucial to prevent bias towards majority classes. While oversampling techniques can mitigate this issue, simple replication may lead to overfitting (Zhou and Sun, 2024; Xue and Zhang, 2016). To overcome this, the Synthetic Minority Over-sampling Technique (SMOTE) algorithm (Chawla et al., 2002) was employed. SMOTE generates synthetic samples for minority classes by interpolating between existing samples, thereby balancing the dataset without introducing redundant data points. The dataset was strictly partitioned into training (80%) and testing (20%) sets using stratified sampling before any data augmentation. The SMOTE was applied exclusively to the training set to balance the class distribution. The testing set remained composed entirely of original samples to reflect real-world scenarios (**Figure 7**). The class distribution of *P. notoginseng* samples before and after SMOTE application is illustrated in **Figure 8**. The 3-grade dataset was balanced to 100 samples per class, while the 5-grade and 6-grade datasets were adjusted to approximately 50 and 45 samples per class, respectively, laying a foundation for the construction of robust model training.

#### 2.3.2 Feature engineering: extraction and selection

Feature engineering plays a pivotal role in enhancing model performance by identifying the most relevant input variables and reducing dimensionality.

As detailed in previous sections, a comprehensive feature set was constructed, comprising: Color Features: 14 dimensions (mean and variance of R, G, B, H, S, a, and b channels). Texture Features (LBP): 59 dimensions (LBP Uniform Pattern). Texture Features (Gabor): 40 dimensions (mean energy of 5 scales × 8 orientations). This resulted in a combined feature vector consisting of all extracted features (All-Features set).

**Figure 9:**
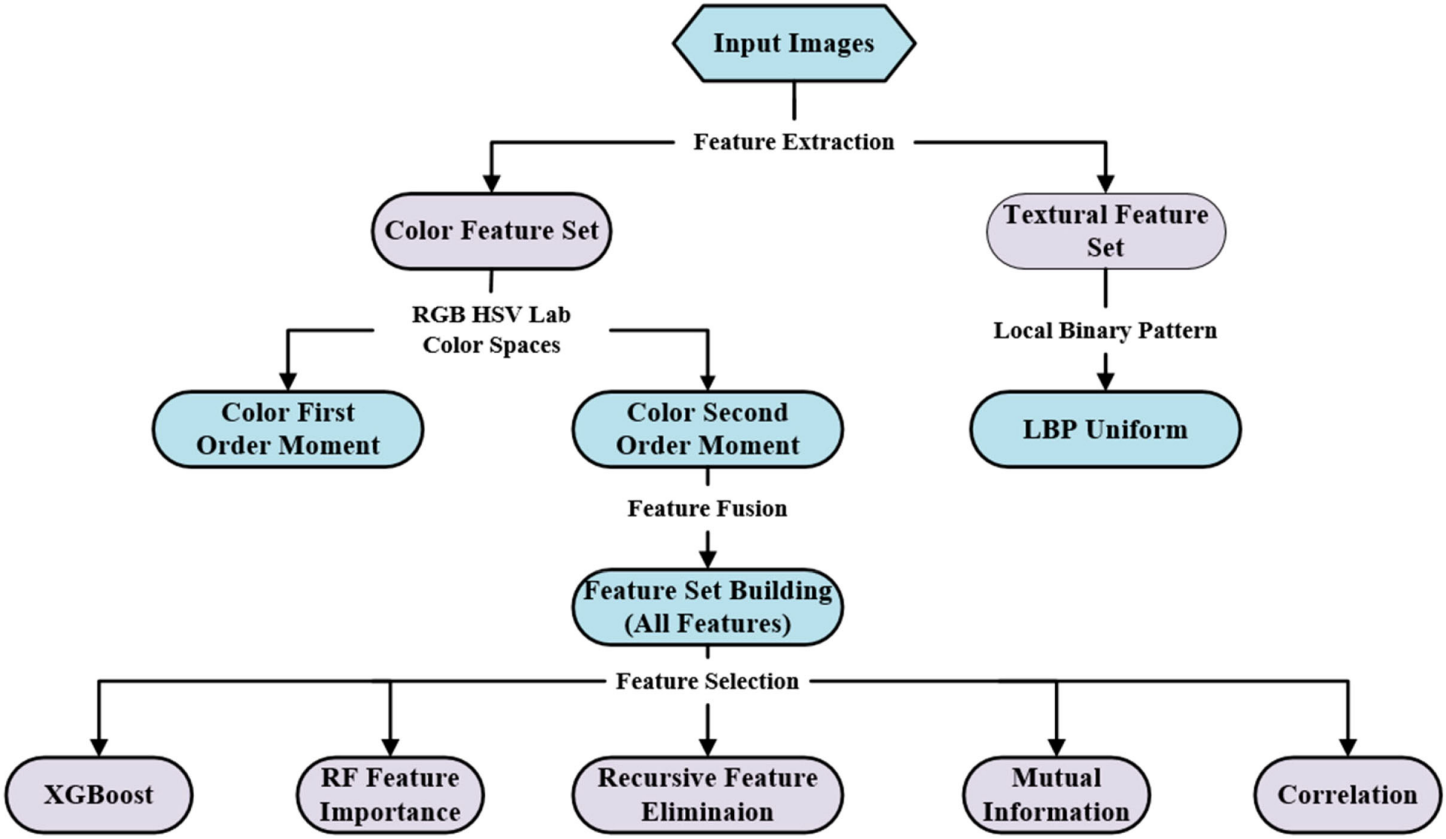
Feature selection flowchart.

To eliminate redundant information and select optimal feature subsets, five distinct feature selection methods were applied to the ‘All-Features’ set: RFI (Random Forest Feature Importance), Cor (Correlation Analysis), MI (Mutual Information), RFE (Recursive Feature Elimination), XGB (XGBoost Feature Importance). For each method, a selection threshold was set to retain variables within the top 60% of cumulative importance. Consequently, six distinct input parameter combinations (the complete ‘All-Features’ set plus the five subsets selected by RFI, Cor, MI, RFE, and XGB) were formed to optimize feature vectors for subsequent model training. The workflow of the feature selection process is depicted in **Figure 9**.

#### 2.3.3 Machine learning methods

Grading models were constructed using four supervised machine learning algorithms implemented in R: CatBoost (Categorical Boosting)、Extra Trees (Extremely Randomized Trees)、XGBoost (eXtreme Gradient Boosting)、LightGBM (Light Gradient Boosting Machine). The training set and test set were split in a 8:2 ratio. The training process involved inputting the six feature parameter combinations (X) into each of these four algorithms. Models were trained to predict the grade of *P. notoginseng* based on three distinct grading criteria (Y: 3-Grade, 5-Grade, and 6-Grade schemes as determined by cluster analysis). This resulted in a total of 60 different model configurations (5 algorithms × 6 feature sets × 3 grading schemes) for comparative analysis. The optimal model for each grading scheme was subsequently selected based on predictive performance.

To quantitatively evaluate the performance of the discriminant models, Accuracy, F1-score, mean average precision (mAP), and Variability were adopted as the evaluation metrics for the models in this study (Vickery, 1979). The calculations are defined as follows (Equations 13–19):

(13) $$Accuracy=\frac{TP+TN}{TP+TN+FP+FN}$$

(14) $$Precision=\frac{TP}{TP+FP}$$

(15) $$Reall=\frac{TP}{TP+FN}$$

(16) $$F1-score=\frac{2\times Precision\times Recall}{Precision+Recall}$$

(17) $$AP=\sum_{k=1}^{N}\left(P\left(k\right)\cdot \Delta R\left(k\right)\right)$$

(18) $$mAP=\frac{1}{C}\sum_{i=1}^{C}AP_{i}$$

(19) $$\sigma =\sqrt{\frac{1}{n-1}{\sum_{i=1}^{C}\left(x_{i}-\bar{x}\right)}^{2}}$$

where TP(True Positives), TN (True Negatives), FP (False Positives), and FN (False Negatives) represent the confusion matrix components. *P(k)*: Precision of the top-k samples, *ΔR(k)*: Change in recall, *C*: Number of classes, *AP_i_​*: Average precision of the *i*-th class.

### 2.4 Validation of the grading system prototype

**Figure 10:**
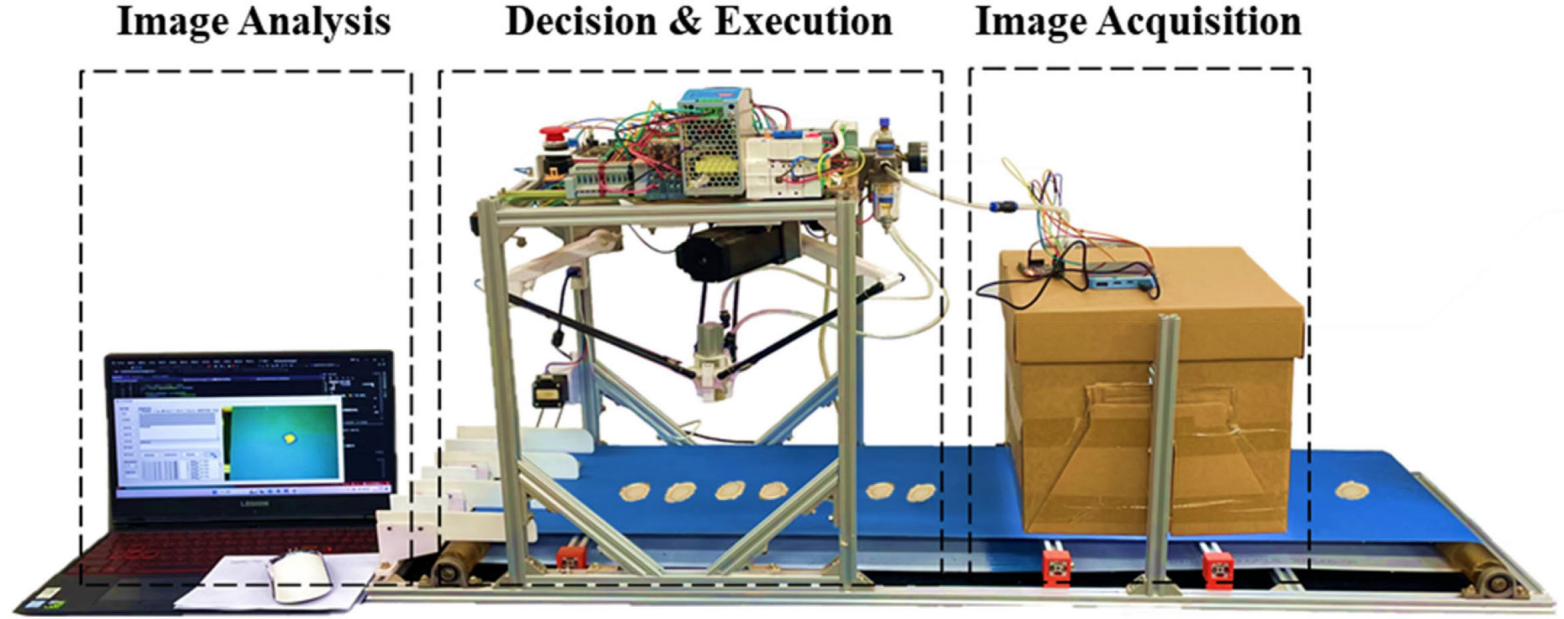
Visual grading device physical drawing.

To validate the practical efficacy of the proposed grading model, a complete prototype visual grading system was constructed (**Figure 10**). The system design adheres to a standard machine vision workflow—encompassing perception, cognition, and action—organized into three functional modules: image acquisition, image analysis, and decision execution. The hardware architecture comprised a host computer, an STM32 microcontroller, an industrial camera, stepper motors driving a conveyor belt, a Delta robotic arm, and power supply units (AC 220V converting to DC 24V/5V).

The operational workflow proceeds as follows: Prior to operation, system initialization involves verifying serial communication between the hardware components and resetting the robotic arm to its home position. Upon initiation, *P. notoginseng* slices are transported via the conveyor belt into the image acquisition zone, where the industrial camera captures high-resolution images. These images are transmitted to the host computer, where the pre-trained grading model extracts features and determines the quality grade of each slice. Subsequently, based on the determined grade, the host computer computes pick-and-place coordinates, performs trajectory planning, and executes inverse kinematics algorithms to derive the necessary joint rotation angles. These parameters are compiled into control commands and transmitted to the STM32 microcontroller. Finally, the microcontroller actuates the Delta robotic arm and gripper to execute specific sorting actions, thereby demonstrating the practical feasibility of the integrated grading system.

## 3 Results and discussion

### 3.1 Establishment of grading criteria

**Figure 11:**
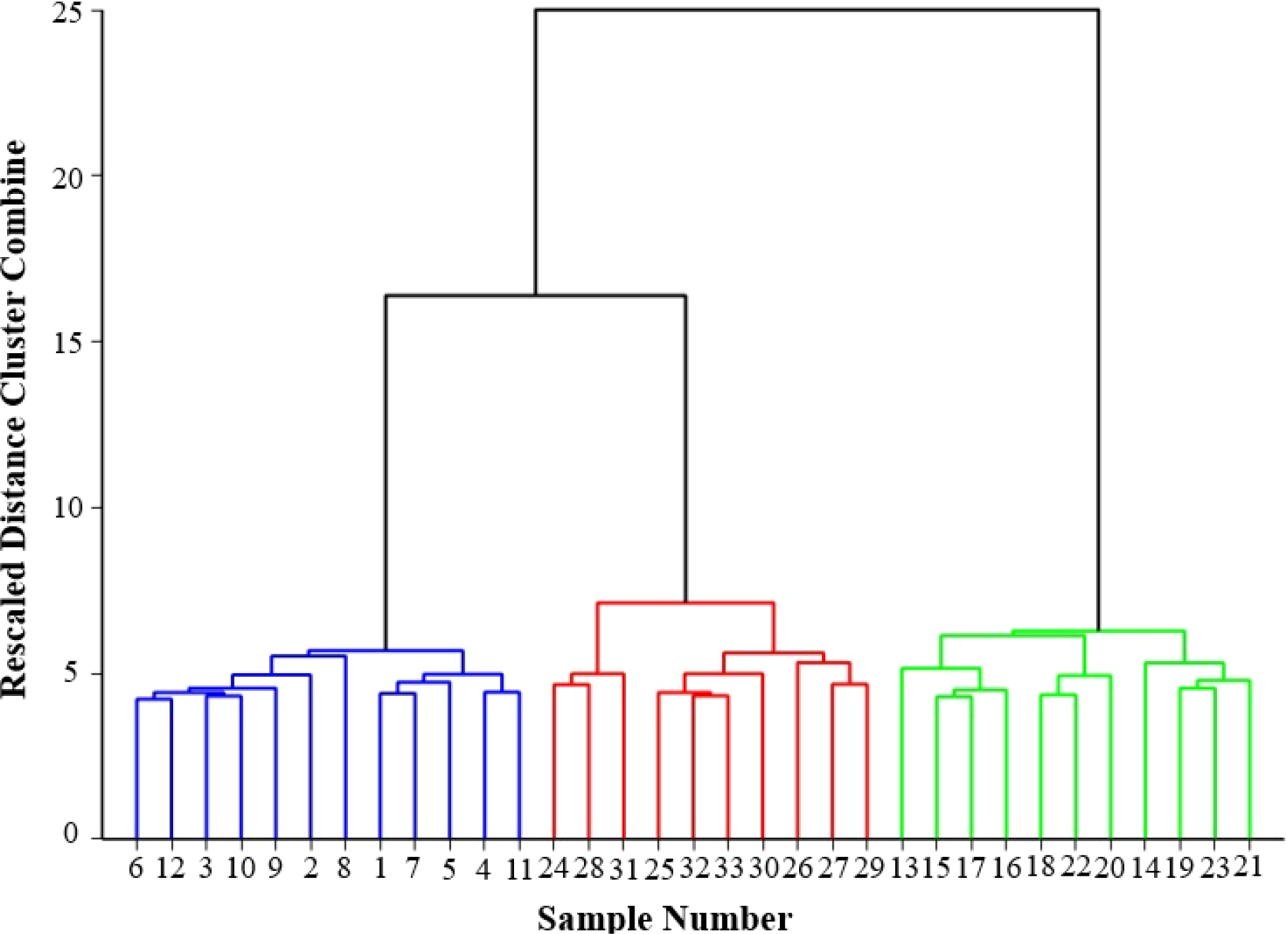
Hierarchical cluster analysis (dendrogram).

To establish objective grading criteria, Hierarchical Cluster Analysis (HCA) was performed using the squared Euclidean distance as the similarity metric and between-groups linkage for cluster merging. The resulting dendrogram (**Figure 11**) visually illustrates the hierarchical aggregation process. As the rescaled linkage distance increases, samples merge into progressively larger clusters. Notably, at higher distance levels, the dendrogram reveals three distinct primary branches (colored blue, red, and green), intuitively reflecting major differences in sample characteristics among the primary categories.

**Figure 12:**
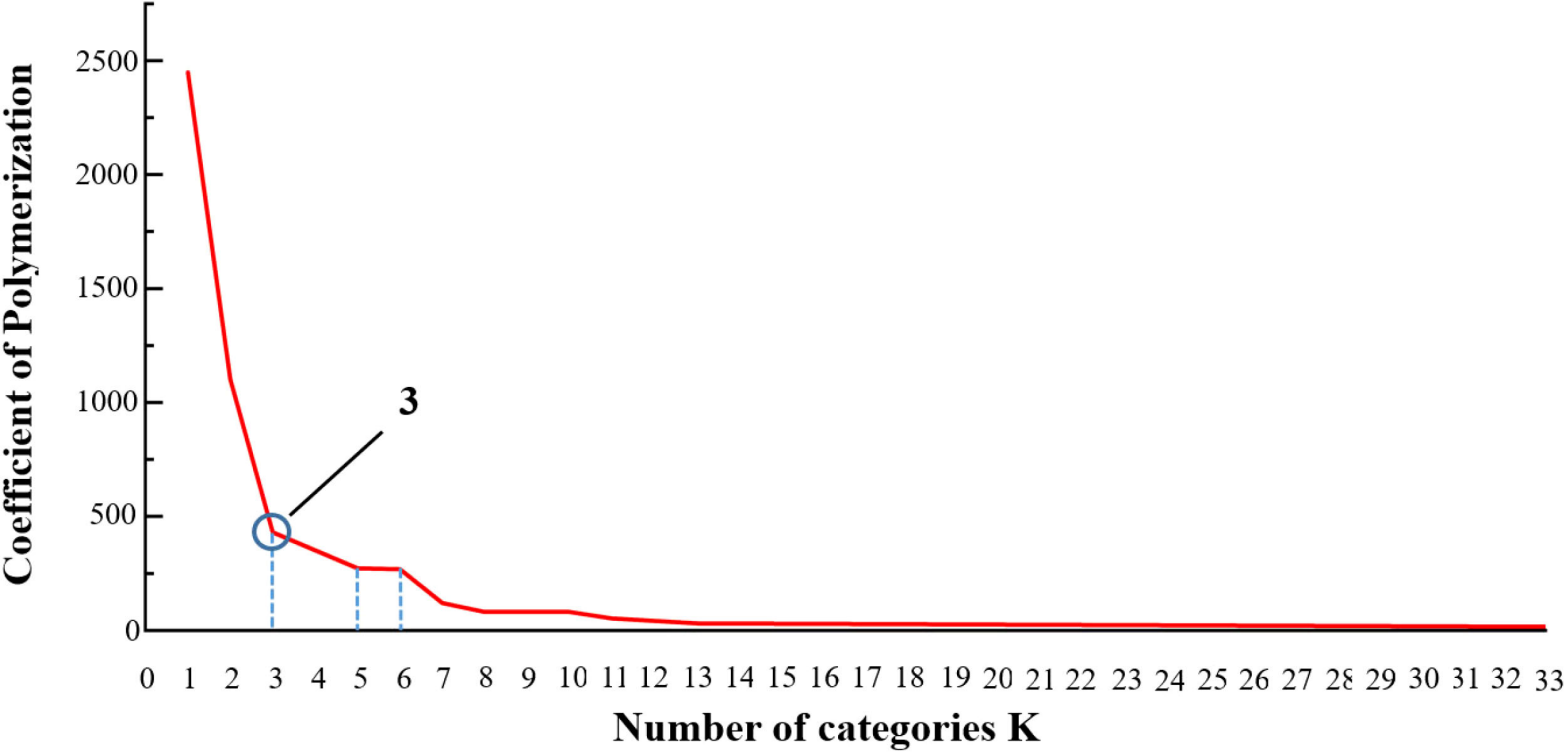
Graph of clustering coefficient.

While the dendrogram provides qualitative insight, the Elbow Method was applied for quantitative determination of the optimal cluster number (*K*). **Figure 12** presents the relationship between *K* and the aggregation coefficient (reflecting clustering compactness). As *K* increases from 1 to 3, the aggregation coefficient experiences a precipitous decline, indicating a significant enhancement in internal cluster similarity. A prominent “elbow point” is observed at K = 3, after which the rate of decline notably plateaus, suggesting K = 3 offers the optimal trade-off between clustering resolution and model simplicity. Furthermore, secondary inflection points are discernible at K = 5 and K = 6, indicating their feasibility as alternative classification granularities. Consequently, aligning these statistical findings with practical application demands for *P. notoginseng* grading, 3-Grade, 5-Grade, and 6-Grade schemes were established as the foundational criteria for subsequent predictive modeling. The PNS content range for each grade as presented in **Table 1**.

**Table 1:** Classification results of grades.

Grade mode	Grade level	Saponin content range (mg/g)
3-Grade	1	54~101
	2	101~129
	3	129~162
5-Grade	1	54~85
	2	85~101
	3	101~129
	4	129~150
	5	150~162
6-Grade	1	54~66
	2	66~85
	3	85~101
	4	101~129
	5	129~150
	6	150~162

### 3.2 Selection of optimal baseline model and performance analysis.

**Figure 13:**
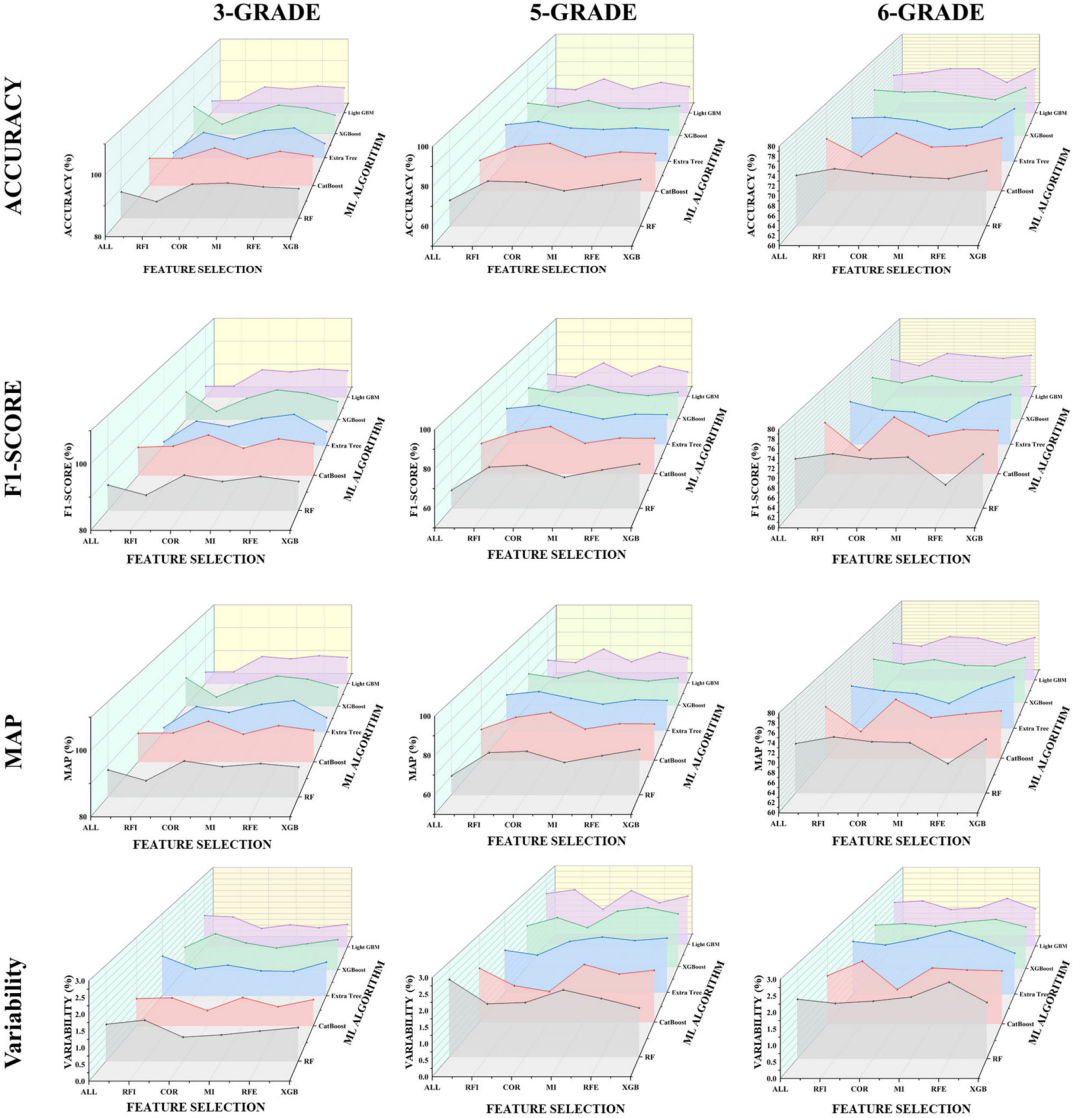
Predictive power of combinations of models.

To determine the most robust strategy for the quality grading of *P. notoginseng* slices, we conducted a systematic evaluation of five machine learning classifiers paired with six feature selection methods. Performance was comprehensively assessed based on Accuracy, F1-Score, mAP, and Variability across three grading granularities (3-Grade, 5-Grade, and 6-Grade). The comparative results, summarized in **Figure 13**, consistently identify the COR-CatBoost combination as the superior baseline model across all metrics.

In the coarse-grained 3-Grade task, COR-CatBoost achieved a peak Accuracy of 93.8% and an F1-Score of 93.8%. Notably, the model exhibited exceptional stability with a Variability of just 0.52%—the lowest among all combinations—alongside a high mAP of 93.9%. While other high-performing combinations, such as RFE-CatBoost (Accuracy: 92.6%) and MI-XGBoost (Accuracy: 92.1%), yielded competitive results, the COR-CatBoost model achieved a 3.8% performance improvement compared to the baseline CatBoost model trained on all features (90.0%).

This superiority becomes even more pronounced in complex fine-grained tasks. For the 5-Grade classification, COR-CatBoost maintained a significant lead with an Accuracy of 77.3%, an F1-Score of 77.2%, and an mAP of 77.5%. This represents a substantial 9.7% improvement over the standard CatBoost model (67.6%) and significantly outperforms the second-best combination, RFI-CatBoost (75.4%). Crucially, the COR-CatBoost model maintained a low Variability of 1.05%, whereas other models exhibited higher fluctuations, suggesting that the COR feature set provides a more consistent signal for the classifier. Similarly, in the most challenging 6-Grade task, the proposed model secured the highest metrics across all categories (Accuracy: 73.2%, F1-Score: 73.1%, mAP: 73.4%). Despite the increased task difficulty, the model demonstrated remarkable robustness with a Variability of 1.18%, proving it to be less susceptible to overfitting compared to the ensemble-heavy Extra Trees and XGBoost variants.

While the COR-CatBoost model demonstrates superior stability and performs effectively in coarse grading, the metrics for the 5-Grade and 6-Grade tasks (ranging from 73.0% to 77.3%) still fall short of the precision required for rigorous clinical and industrial quality control standards (typically >85%). This performance ceiling suggests that while the model is robust (exhibiting low variability), the use of default hyperparameters limits its capacity to fully capture the non-linear boundaries between adjacent, high-similarity grades. Consequently, further hyperparameter optimization is necessary to enhance classification precision.

### 3.3 Model parameter optimization

**Figure 14:**
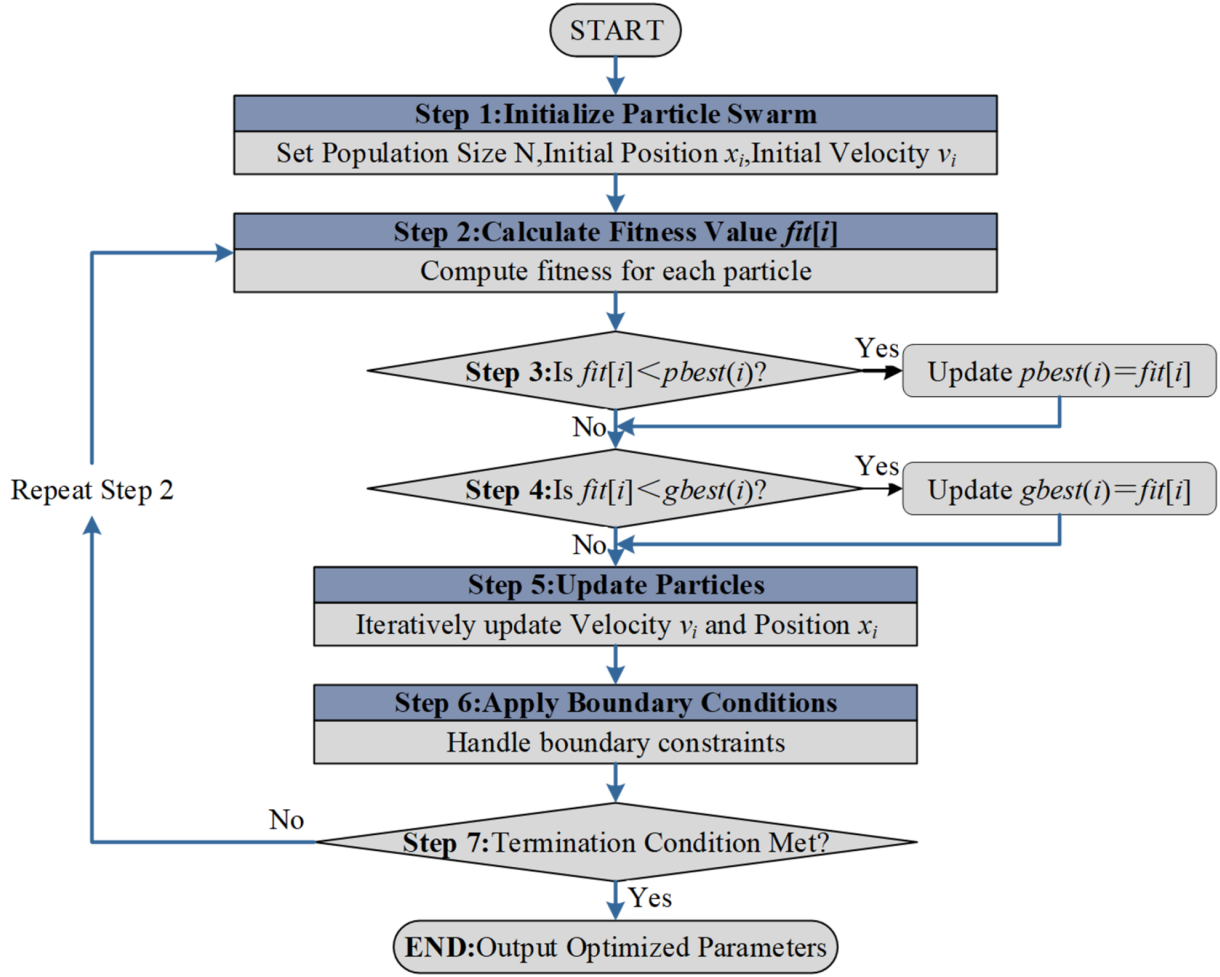
PSO algorithm flow.

To strictly align the model with the complex feature space of the samples, hyperparameter optimization was implemented using the Particle Swarm Optimization (PSO) algorithm. The detailed optimization steps are illustrated in **Figure 14**.In this study, the optimization focused on four critical hyperparameters of the CatBoost algorithm: learning rate, depth (depth of the tree), iterations (number of trees), and l2_leaf_reg (L2 regularization coefficient). The optimization ranges were set as follows: learning rate [0.001, 0.1], depth [4, 12], iterations [500, 2000], and l2_leaf_reg [1, 10]. The classification accuracy on the validation set served as the fitness function for the PSO algorithm.

Complex machine learning models often suffer from hyperparameter sensitivity. Instead of employing traditional grid search which is computationally expensive and prone to local optima, the PSO strategy was integrated. This decision was driven by PSO’s ability to efficiently navigate the high-dimensional parameter space of the CatBoost model, ensuring a balance between model complexity and generalization capability.

Applying these optimized parameters yielded substantial improvements in model performance across all grading granularities on the test set. **Table 2** presents the comparison between the default and PSO-optimized models. For the coarse-grained 3-Grade classification, the optimized COR-CatBoost model achieved a near-perfect Accuracy of 98.6% and an F1-score of 98.5%, representing an improvement of 4.8% over the baseline. More significantly, the optimization successfully addressed the bottlenecks in fine-grained grading. For the 5-Grade classification, the Accuracy surged from 77.3% to 88.2%, with the F1-score improving to 87.9%. Similarly, for the most challenging 6-Grade classification, the model achieved an Accuracy of 84.5% and an F1-score of 84.1%, marking an 11.3% increase in accuracy compared to the unoptimized baseline. These results indicate that the PSO algorithm effectively navigated the parameter space to find a global optimum, significantly enhancing the model’s ability to discriminate between high-similarity grades. The final COR-CatBoost-PSO model demonstrates high precision and robustness, fully meeting the requirements for rapid, non-destructive quality evaluation of *P. notoginseng*.

**Table 2:** Optimized model parameters.

Name	Value
Training Time	37 min 28.39 s
Base Learner	dart
Number of Base Learners	199
Learning Rate	0.0188
L1 Regularization Term	0.0071
L2 Regularization Term	0.7223
Sample Subsampling Rate	1
Feature Subsampling Rate	1
Node Split Threshold	0
Minimum Weight of Samples in Leaf Nodes	0
Maximum Depth of Tree	12
Minimum Number of Samples in Leaf Nodes	10

### 3.4 Model validation

To verify the applicability, generalization ability, and effectiveness of the proposed *P. notoginseng* grading method, a dual-stage validation strategy was employed. This strategy encompassed 10-fold cross-validation to assess algorithmic robustness and a physical bench test to evaluate industrial feasibility.

#### 3.4.1 10-fold cross-validation for model robustness

Prior to the physical bench test, 10-fold cross-validation was implemented on the optimized COR-CatBoost-PSO model to comprehensively evaluate its generalization performance and mitigate selection bias associated with a single training-test split. The SMOTE-balanced dataset was randomly partitioned into ten mutually exclusive subsets of equal size. In each iteration, nine subsets constituted the training set for hyperparameter fine-tuning (**Table 3**), while the remaining subset served as the independent test set. This process was repeated ten times, ensuring each subset functioned as the test set exactly once.

**Table 3:** 10-fold cross-validation performance of COR-CatBoost-PSO model.

Grading scheme	Accuracy (mean ± SD, %)	MAP (mean ± SD, %)	Variability (mean ± SD, %)	F1-score (mean ± SD, %)
3-Grade	98.2 ± 0.5	97.9 ± 0.7	0.6 ± 0.2	98.1 ± 0.5
5-Grade	87.8 ± 1.2	87.2 ± 1.1	1.3 ± 0.4	87.6 ± 1.2
6-Grade	83.9 ± 1.5	82.8 ± 1.4	2.1 ± 0.5	83.6 ± 1.5

Key performance metrics—Accuracy, F1-score, mean average precision (mAP), and Variability—were calculated for each fold. The mean and standard deviation across the ten iterations served as the final performance indicators. As detailed in **Table 3**, the COR-CatBoost-PSO model demonstrated exceptional stability and generalization: the mean accuracy for 3-Grade, 5-Grade, and 6-Grade classifications reached 98.2 ± 0.5%, 87.8 ± 1.2%, and 83.9 ± 1.5%, respectively. The low variability (≤2.1%) and high mAP (≥82.8%) across all schemes indicate that the model is insensitive to random dataset partitioning. These results confirm the model’s strong anti-interference capability, establishing a robust algorithmic basis for the subsequent physical bench test.

#### 3.4.2 Physical bench test for industrial application feasibility

**Figure 15:**
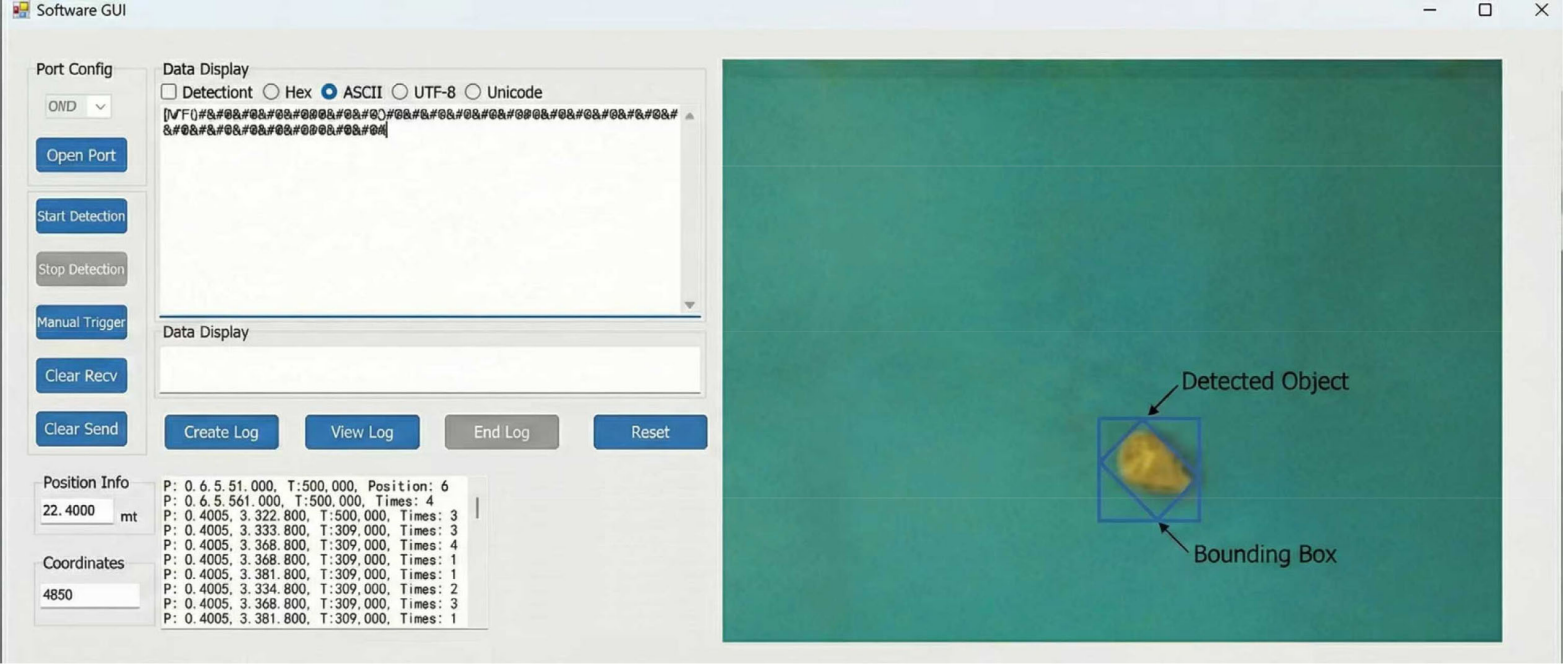
Graded testing system.

Building upon the robust results of the cross-validation, a physical bench test was conducted using 50 independent samples collected from Wenshan. These samples were distinct from those used in model training and validation to ensure testing objectivity. Sample processing and physicochemical detection strictly adhered to the protocols outlined in Sections 2.1 and 2.2. The test was executed on the custom-built integrated visual grading device (**Figure 10**), with the detection interface displayed in **Figure 15**.

The results indicated that the system achieved a 100% Classification Accuracy for *P. notoginseng* slices, with zero false positives or negatives. The Delta robotic arm demonstrated high precision, with an average positioning error of only 1.2 mm, fully meeting industrial grading requirements. Furthermore, the processing time per slice ranged from 0.8 to 1.5 s, realizing rapid, non-destructive grading. Integrating the findings from both the 10-fold cross-validation and the physical bench test, it is evident that the proposed COR-CatBoost-PSO model possesses not only superior algorithmic robustness but also practical viability for industrial deployment. This seamless integration of algorithm optimization and engineering application provides a high-precision, high-efficiency solution for the intelligent and standardized quality control of *P. notoginseng*.

### 3.5 Discussion

Our findings align with and extend recent advancements in the intelligent grading of TCM. For instance, Huang and Xu (2023) demonstrated the feasibility of automated sorting using standard machine learning models. However, their approach was primarily limited to coarse-grained classification and struggled with high-dimensional class overlap, leading to reduced accuracy in fine-grained tasks. In contrast, our proposed COR-CatBoost-PSO framework explicitly addresses these challenges. By integrating Pearson correlation analysis (COR) for feature dimensionality reduction, we effectively filtered out redundant visual information. Furthermore, the introduction of the PSO algorithm allowed the model to escape local optima during hyperparameter tuning. This synergy explains why our model maintained high accuracy (83.9%) even in the complex 6-Grade scenario, where the boundaries between saponin content levels are inherently non-linear and subtle. The bench test results (0.8–1.5 s per slice) further confirm that this algorithmic complexity does not compromise industrial processing speed.

Despite the promising results, this study is not without limitations, which warrant critical attention. Firstly, the dataset utilized in this study, comprising 143 samples, is relatively modest for training complex machine learning models. While 10-fold cross-validation and variability analysis verified the model’s robustness, the lack of a massive, multi-origin dataset may limit the model’s generalization capability when applied to *P. notoginseng* from widely differing geographical regions or harvest seasons. Secondly, the image acquisition in this experiment was conducted under strictly controlled laboratory lighting. In real-world industrial environments, fluctuations in ambient light and the presence of dust could introduce noise to the visual features. Future work needs to incorporate data augmentation or transfer learning strategies to enhance the model’s anti-interference ability against environmental variations.

## 4 Conclusions

In this study, a rapid and non-destructive quality grading method for *P. notoginseng* slices was successfully developed by integrating machine vision technology with chemometrics. The main conclusions are as follows:

1. By determining the PNS content of 143 sample batches via HPLC and applying HCA with the Elbow Rule, the slices were scientifically categorized into 3-Grade, 5-Grade, and 6-Grade classes. This provides a rigorous data-driven foundation for investigating the correlation between external appearance and internal quality.
2. Through a systematic comparison of five machine learning classifiers and six feature selection strategies, the COR-CatBoost combination was identified as the superior baseline model. The 10-fold cross-validation strategy demonstrated that the model achieved mean accuracies of 98.2% ± 0.5%, 87.8% ± 1.2%, and 83.9% ± 1.5% for the 3-Grade, 5-Grade, and 6-Grade classification tasks, respectively. Furthermore, low variability (0.6%–2.1%) was observed across all grading schemes.
3. PSO algorithm was introduced to globally optimize the hyperparameters of the COR-CatBoost model. T The optimized COR-CatBoost-PSO model, with a training time of 37 minutes 28.39 seconds, adopted dart as the base learner, a learning rate of 0.0188, an L1 regularization term of 0.0071, an L2 regularization term of 0.7223, and a maximum tree depth of 12. It achieved significant performance improvements, with final accuracies reaching 98.6%, 88.2%, and 84.5% for the 3-Grade, 5-Grade, and 6-Grade schemes, respectively. These results confirm that the proposed method meets the precision requirements for industrial applications, offering a highly efficient and objective solution for the intelligent quality control of traditional Chinese medicine.

Despite the demonstrated efficacy of the proposed model, the current grading method predominantly relies on the content of PNS as the sole critical indicator. The intrinsic mechanisms linking external visual representations to the comprehensive quality attributes of *P. notoginseng* remain to be fully elucidated. Consequently, future investigations should focus on decoding the complex mapping relationships between the macroscopic morphological features (specifically color and texture) of the slices and their broader physicochemical and physiological properties. Establishing a multi-dimensional quality evaluation system that bridges visual perception with internal bioactivity will be a core direction for subsequent research, thereby enhancing the interpretability and applicability of machine vision in Traditional Chinese Medicine.

## Data Availability

The raw data supporting the conclusions of this article will be made available by the authors, without undue reservation.
